# Transparency in Negotiation of European Union With Big Pharma on COVID-19 Vaccines

**DOI:** 10.3389/fpubh.2021.647955

**Published:** 2021-02-18

**Authors:** Salvatore Sciacchitano, Armando Bartolazzi

**Affiliations:** ^1^Department of Clinical and Molecular Medicine, Sapienza University, Rome, Italy; ^2^Laboratory of Biomedical Research, Niccolò Cusano University Foundation, Rome, Italy; ^3^Laboratory of Surgical and Experimental Pathology, St Andrea University Hospital, Rome, Italy; ^4^Department of Oncology-Pathology, Cancer Center Karolinska Universitetssjukhuset Solna, Stockholm, Sweden

**Keywords:** COVID-19, vaccines, transparency in negotiation, big pharmaceutical companies, advance purchase agreements

## Introduction

Immunization through vaccination represents one of the most cost-effective public health interventions and the main tool for primary prevention of communicable diseases. Vaccination programs and vaccine prices, however, vary considerably among and within countries in the European Union (EU), because of the differences in the way healthcare systems are organized at the national or regional levels. These differences may lead to a new threat represented by the so-called “vaccine nationalism” that keep negotiations with the pharmaceutical industry behind the closed doors of each single nation, thus undermining global efforts to ensure fair access to vaccines for everyone (1). The severity of the recent COVID-19 pandemic is urging a major change in our capabilities to respond in the most appropriate and coordinated manner to the emergency situation. Transparency about the different roles of all stakeholders, either public or private, of vaccine manufacturers, and of health authorities and, most importantly, transparency in negotiations regarding vaccine price, could help avoid misconceptions, thus strengthening the collaboration required to protect against the pandemic.

## Vaccine Price

New vaccine pricing is a complicated process, including target population analysis, mapping of potential competitors, quantification of the incremental value, determination of the vaccine positioning in the marketplace, assessment of the vaccine price-demand curve, calculation of the costs of manufacturing, distribution, research and development, and inclusion of the various legal and regulatory expenses (2). The effective final price of the new vaccine may, eventually, be different for different purchasers because of various discounts, promotions, and incentives that the manufacturers may apply considering geographic and economical situations, as well as different times of the year, especially for flu vaccines (3). Transparency in the negotiation for vaccine prices has been a matter of debate for many years. In 2014, WHO launched the vaccine product, price, and procurement initiative, named Market Information for Access to vaccines (MI4A), aimed to improve vaccine price transparency (4). Thanks to the database created by the MI4A and improved price transparency, many low- or middle-income countries increased their possibility to access information, their capacity to negotiate affordable prices and strengthen their access to affordable vaccines (5). However, the issue is still far from being resolved.

## The Lesson (Unlearned) from FLU Vaccine

The emergence and subsequent global spread of the 2009 A(H1N1) influenza, also known as swine flu, with nearly 2,000 deaths in the EU, prompted health authorities around the world to review their response and to improve the reaction to the pandemic. During the 2009 pandemic, vaccine manufacturers greatly increased influenza vaccine production capacity and adopted a “tiered-pricing” strategy, where the price of a vaccine was mainly based on the level of income of the country (6). At that time EU member states struggled to obtain sufficient quantities of vaccines as quickly as needed and had to accept unfavorable contractual terms (7). The most developed countries placed large advance orders for the 2009-H1N1 vaccine and bought virtually all of what the vaccine companies could manufacture. National interests clearly prevailed over global solidarity. Wealthier governments that had provisional contracts with vaccine makers monopolized the global vaccine supply. By means of such contractual obligations, manufacturers committed all their capacity to produce and deliver vaccines to those who could pay the most (8). As a result, the 2009-H1N1 vaccine production affected the amount and timing of vaccines available for developing countries. Even though WHO entered talks with manufacturers and developed-country governments to secure some vaccines for developing countries through monetary donations both from manufacturers and developed countries, such donations still left the developing world with limited supplies or the vaccines arrived too late to be of much benefit. However, the impact of the H1N1 virus was less severe than anticipated, and health authorities of many countries had to face the problem of stockpiles of unnecessary swine flu vaccines. They had to negotiate with manufacturers over the suspension of delivery for surplus vaccines, and they tried to sell or donate at least part of them. The experience with previous pandemic flu prompted the manufacturers and the health authorities to work together to enhance global access, and to strengthen future preparedness. In 2018, a multidisciplinary expert panel was invited by the EU to identify measures and actions to improve vaccination coverage and to encourage close cooperation and better integration of public health and primary care services among member states in the EU^1^. Among the changes proposed, there were some crucial scientific and technical improvements to rapidly select optimal vaccine viruses, actions to speed up vaccine production, and instruments to implement vaccine supply by means of the establishment of appropriate agreements prior to a pandemic.

However, was that experience useful in improving our ability to combat the actual COVID-19 pandemic? Are we facing a replay of the past H1N1 influenza pandemic of 2009, with wealthy countries hoarding the vaccines? A concern was raised regarding transparency of the different roles of all stakeholders and about price, liability, and availability of vaccines. Full transparency of the vaccines' contracts, as well as the publication of clinical trials data before marketing authorizations are granted, is requested and this represents the key to widespread use of potentially life-saving vaccines.

## The COVID-19 Pandemic

The global COVID-19 pandemic has stricken the EU with almost 17 million people infected and more than 400,000 deaths as of data obtained on week 1 of 2021 by the European Center for Disease Prevention and Control. There is a global request for a safe and effective vaccine against COVID-19 (9). The urgency to manufacture and to make accessible to everyone a successful COVID-19 vaccine prompted the EU to promote a common strategy (EU Com. n. 2020/245). In this regard, the COVID-19 pandemic is accelerating the interdependence of all EU economies and societies to form a closely integrated single market, as indicated by the 8th President of the EU commission, Jacques Delors, who launched this program in 1985, allowing a joint action at EU level on health policies, including the market for drugs and vaccines. This represents an excellent opportunity to be one step closer toward the unification of the different national health policies, thus eliminating unjustifiable functional duplications between the European Medicines Agency (EMA) and every single national drug agency, at least regarding negotiation procedures.

## The EU Strategy for COVID-19 Vaccines

According to the program for the years 2014–2020, the EU's action in the field of health was to complement and support national health policies, encourage cooperation, and promote coordination between their programs, in full respect of the responsibilities of each single member state for the definition of their health policies and the organization and delivery of health services and medical care (EU Reg. n. 2014/282). Following the unprecedented public health emergency created by COVID-19, the EU has modified the previous choice of not defining any specific health policies, and a range of measures have been taken by the EMA and by a network of national competent authorities to facilitate, support, and speed up the development and marketing authorization of treatments and vaccines (EU Reg. n. 2020/1043). A new program, named the EU4Health program, has been approved for the years 2020–2021, with the aim of strengthening the EU's role on health, and its capacity to react, manage, and coordinate its powers by means of a “European Union of Health” (EU Com. n. 2020/405). The new EU strategy for COVID-19 vaccines was presented in June 2020 (EU Com. n. 2020/245). It consisted of three objectives: (i) ensuring the quality, safety, and efficacy of vaccines; (ii) securing timely access to vaccines for member states and their population, while leading a global solidarity effort; and iii) ensuring equitable access for all to an affordable vaccine as early as possible. Such a strategy focused on the production and on the procurement of sufficient doses of vaccines for each member state, through Advance Purchase Agreements (APAs) negotiated with vaccine producers. Legal instruments to support such emergency action were established in 2016 (EU Reg. n. 2016/369) and amended in 2020 (EU Reg. n. 2020/521). Based on the considerable legal and practical difficulties in purchasing supplies or services in emergency situations by the contracting authorities from each member states, the EU commission extended its possibilities to purchase supplies or services on behalf of them and advocated the authority to directly negotiate for the purchase of health supplies and, particularly, of COVID-19 vaccines, to get maximum benefit in terms of economies of scale and risk–benefit sharing.

## The EU Position on Transparency When Negotiating Advance Purchase Agreements

According to these emergency regulations, a number of derogations from previous articles have been set out and applied for a limited period of time, from February 1, 2020 until January 31, 2022. In no document, however, was a derogation from the transparency on negotiations of APAs for COVID-19 vaccines reported. In a statement to the plenary of the EU Parliament on transparency of purchase as well as access to COVID-19 vaccinations, released by Mrs. Stella Kyriakides, commissioner on health and food safety, it was reported that “*vaccinations, once we have a vaccine which is proven safe and effective, will play a crucial role: in saving lives, in containing the pandemic, in protecting health care systems, in helping to restore our economy*” (statement by Kyriakides, 12.11.2020). The EU commission has worked intensively to have a common EU portfolio of different vaccines against COVID-19 as diverse as possible. Many APAs have already been signed with *Johnson & Johnson, AstraZeneca, Sanofi-GSK, Janssen Pharmaceutica NV, BioNtech/Pfizer, CureVac, and Moderna*. To date, the commission has secured at least 1.2 billion doses and has fulfilled its commitment of ensuring equitable access to “*safe, effective, and affordable vaccines*.” It appears clear that such a huge number of doses will represent a relevant cost for the EU health system, and negotiations for the price of each single vaccine is a significant matter of debate. Following the EU commission negotiations, the Italian ministry of health has launched its vaccine strategy plan aimed to ensure 202.5 million doses for all Italian people (strategic plan for vaccine anti-SARS-CoV-2/COVID-19, updated on 15.12.2020). Centralized negotiation procedures have obvious advantages; however, they demand transparency, especially when they involve huge public financial resources. It is therefore expected that the EU commission maintains a high level of accountability and transparency, and it is reasonable to ask what procurement rules are being followed and how the professionals involved were recruited. In her statement Mrs. Stella Kyriakides recognizes the importance of transparency. However, she admits that “*due to the highly competitive nature of this global market, the commission is legally not able to disclose the information contained in the contracts*.” It is a special request by the companies, in fact, that “*such sensitive business information remains confidential between the signatories of the contract.”* The commission, therefore, cannot decide to unilaterally disclose the terms of negotiation without the consent of all involved parties.

## The Position of the Pharmaceutical Companies

There are many requests, coming from several different sources, directed to the pharmaceutical corporations to open their books to show the economic aspects of the contract, the costs of vaccine production, and how much the countries agreed to pay for each vaccine type. The major concern is that wealthy countries could buy up huge amounts of vaccine stocks, leaving poorer countries facing huge difficulties to afford what they need. The major pharmaceutical companies, represented by the International Federation of Pharmaceutical Manufacturers and Associations (IFPMA) and by the European Federation of Pharmaceutical Industries and Associations (EFPIA), respond that they are committed to working with governments, partners, and payers to ensure that vaccines will be available and affordable for people at a fair and reasonable price. In addition, following the EMA initiative, they issued a joint pledge promising to implement extraordinary transparency measures in the context of COVID-19 (10). Such measures include speeding up the publication of key documents, accelerating the announcements of drugs included in the compassionate use programs, implementing earlier deadlines for publishing public evaluation reports, publishing the complete version of the management plan as well as the clinical trial data, while also protecting privacy rights. Although such an initiative will undoubtfully have advantages in transparency for healthcare professionals, researchers, media, policymakers, and the general public, they are focused on regulatory processes and procedures for patients, and contain no mention concerning transparency in the negotiation procedures. According to the pharmaceutical companies, non-disclosure clauses are a standard feature in APAs. They are necessary to protect sensitive negotiations and business-related information, including financial information, development, and production plans. The two pharmaceutical companies *Moderna* and *Pfizer* do not hide that they would be making a profit on their vaccines. *Pfizer* CEO Albert Bourla said to Barron's magazine in July 2020 that since the private sector found the solution for diagnostics and, again, since the private sector found the solution for therapies and vaccines, it is wrong to think that the private sector should not be making a profit on the drugs and vaccines they introduce to fight COVID-19 (11). This is frustrating when we consider that there is a huge amount of public investment behind the contracts for COVID-19 vaccines. This may represent a huge privatization of public money. On the other side, *Johnson & Johnson* and *AstraZeneca* indicated that they would sell vaccines at their cost through the pandemic. Recently, *Johnson & Johnson* announced an agreement in principle with the Global Alliance for Vaccines and Immunization (GAVI Alliance) to supply *Janssen*'s COVID-19 vaccine to lower-income countries in 2021 (12). *Glaxo* and *Sanofi* also declared that they do not expect to profit during the pandemic phase (13).

## Transparency in the Negotiations for COVID-19 Vaccines

By advocating the authority to directly negotiate for the purchase of health supplies and, particularly, of COVID-19 vaccines, the EU derogated from this previous commitment to respect the responsibilities of each single member state for the definition of their health policies. This is justified by the emergency created by the COVID-19 pandemic. However, should transparency on negotiations for COVID-19 vaccines be derogated as well? Why has the commission accepted to be legally bound to secrecy and decided to forgo its duties in accountability and transparency to the people it is supposed to serve? Have all the potential long-term consequences of this secrecy on the EU pharmaceutical market been considered, and on what basis was it decided to accept this secrecy using public funds without seeking public consent? Vaccine pricing differs widely among countries, and a global approach has been advocated to guarantee that all subjects can be vaccinated, especially those of low-income countries (14). Many relevant concerns have been raised about the new COVID-19 vaccines (15). We believe it is relevant to answer another key question: Is transparency in the negotiations of health products still a priority issue? It certainly was in 1988, when the EU council mandated a specific directive on this topic (L40/8, 89/105/EEC). In 2018, WHO published its draft road map for access to medicines, vaccines, and other health products 2019–2023, encouraging exchanges of information and knowledge among different countries and supporting a global and regional collaboration to increase price transparency for quality-assured health products (WHO, 144th session, Provisional agenda item 5.7, EB144/17). Transparency in the negotiations on COVID-19 vaccines has been advocated by many (16, 17). One of the most active medical humanitarian organizations, Médecins Sans Frontières, requested both transparency on how public money is handed over to pharma corporations (18) and recommended accessibility with equity for everyone who needs COVID-19 vaccines. The international non-governmental organization Human Rights Watch focused attention on “opaque” vaccine deals that could undermine the global recovery from the pandemic and claiming that “health not wealth” should determine access to a COVID-19 vaccine. The transparency issue was raised again in 2019, at the 72nd World Health Assembly, in Geneva by former representatives of the Italian Ministry of Health and the former director general of AIFA, Dr. Luca Li Bassi, in a resolution for transparency when negotiating drug prices (WHA Doc. 72.8/2019). The aim was to promote reforms in national, European, and global frameworks to make quality medicines, vaccines, diagnostic tests, and new medical technologies and therapies available and affordable. For his work, Li Bassi was awarded the 2019 “International Transparency in Medicines Policies Awards” by the French Civil Society watchdog group l'Observatoire Médicaments Transparences (the Observatory for Transparency in Medicines). Another step ahead toward transparency on negotiation for COVID-19 vaccines was recently made by the Brazilian public research institution, Fundação Oswaldo Cruz (Fiocruz), who disclosed the terms of its agreement with *AstraZeneca* for the production of a potential future COVID-19 vaccine^2^. Despite all these initiatives, transparency in the EU negotiation of the COVID-19 vaccines is still lacking. Recently, even members of the EU parliament (MEPs) called for more clarity and transparency on COVID-19 vaccine contracts and asked to grant access to all the APAs for COVID-19 vaccines. Therefore, even MEPs do not have access to the most basic information, such as: how much will the production of these vaccines cost? and what will be the liability of the companies for any damage caused by a vaccine? A partial positive response was given by Mrs Sandra Gallina, the EU's lead negotiator on COVID-19 vaccine contracts. She opened a dedicated “reading room,” that currently only contains the contract with *CureVac*, to allow a select few MEPs to review the redacted versions of the contract, signed with companies. We believe that this is not enough, and persistence of secrecy in legal agreements by the EU and vaccine manufacturers represents a barrier to global equitable COVID-19 vaccine access and distribution (19). We, therefore, support the request, recently posted by 39 civil society organizations, including the European Public Health Alliance, and directed to the EU commission and to the EU national governments to ensure a maximum degree of transparency in the EU's exchanges, negotiations, and deals with pharmaceutical companies over COVID-19 vaccines^3^.

## COVID-19 Vaccine Price Leaks

In December 2020, documents relating to COVID-19 vaccines and, in particular, to one from *Pfizer/BioNTech* were stolen from the EMA agency, which, after Brexit, is located in the Netherlands. EMA confirmed the cyber-attack, and criminal investigations are ongoing to clarify whether the stolen data are up for sale or if they have been published for anyone to access.

However, this is not only a case of leaking information regarding COVID-19 vaccines. The COVID-19 vaccine prices that the EU commission kept secret and covered by “confidentiality” were released via Twitter, seemingly in a blunder, by Belgium's budget state secretary, Eva De Bleeker. She tweeted the price of all the COVID-19 vaccines that the EU had negotiated with pharmaceutical companies on behalf of its 27 member states, with the list of the country's number of vaccines and the price they were paying per each dose. The tweet was quickly removed, but the list had already been made public, and it was reported by the New York Times (20). The pricing data contained in the list were not confirmed by the EU spokesman, who declared that the secrecy about the prices paid by the EU is legitimate and is part of the negotiation for the vaccine. It is likely that such information on COVID-19 vaccines prices will influence future negotiations with manufacturers. According to such leaked information, the United States, who negotiated prices and arranged to buy doses for every American directly, is paying more than Europe. In any case, it is relevant to mention that during these days, all the hospitals that operate in the United States have been required to comply with the centers for medicare and medicaid services' price transparency requirements detail, so-called “the Rule.” They are required to make public a list of their standard charges for the services they provide^4^. According to COVID-19 vaccine policies and guidance, “the Rule” also includes the price of COVID-19 vaccines, not only for medicare but also for medicaid services as well as for private insurance.

## Consequences of the Absence of Transparency on COVID-19 Vaccine Negotiations

**Figure 1:**
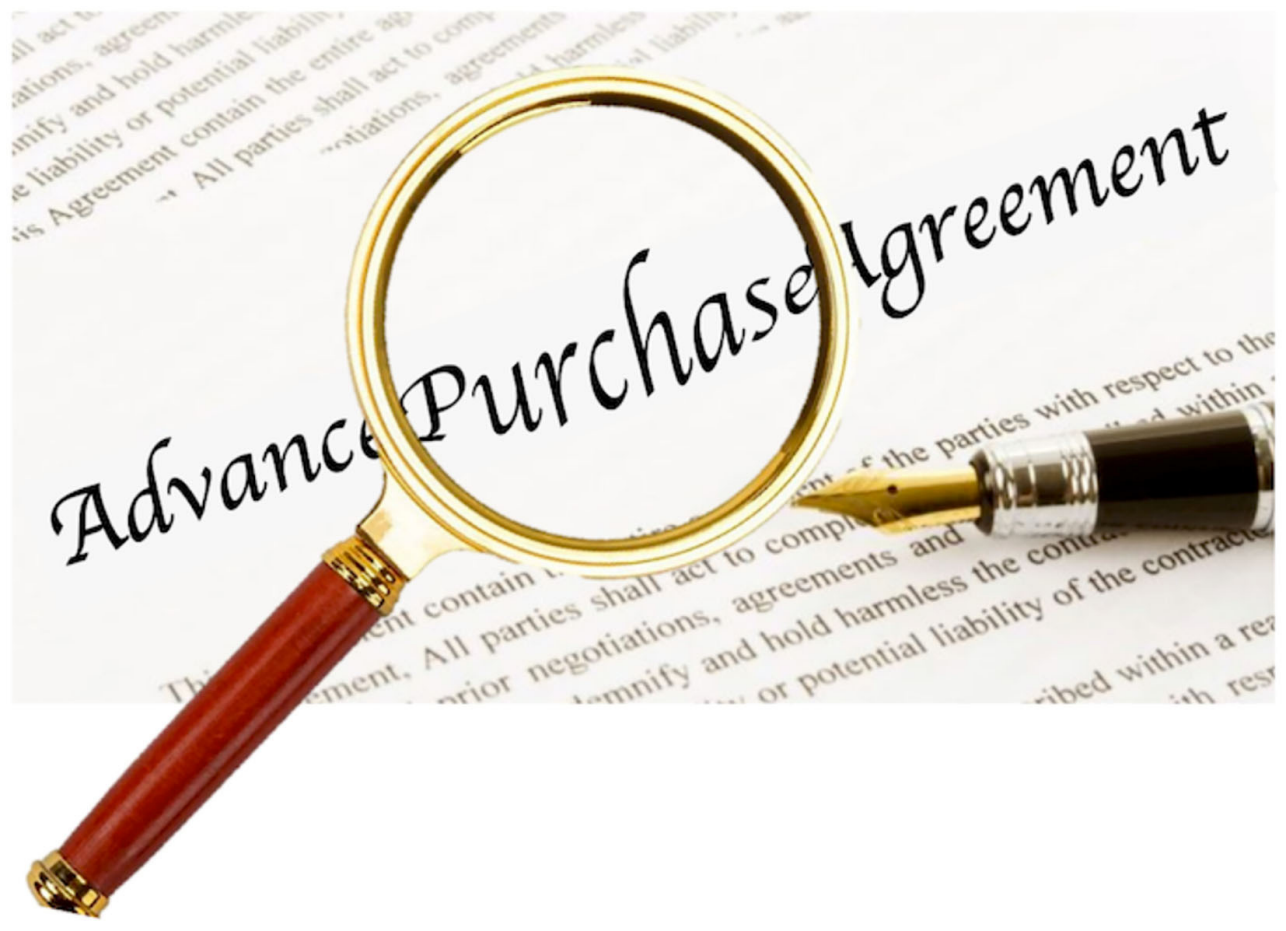
Transparency in the negotiations for Advance Purchase Agreements (APAs) on COVID-19 vaccines. The EU is coordinating a joint effort to secure the acquisition of a sufficient quantity of COVID-19 vaccines in the EU through Advance Purchase Agreements (APAs) with vaccine producers, but transparency in negotiations is lacking, and sensitive business information remains confidential between the signatories of the contract.

The absence of transparency on the negotiation for COVID-19 vaccines frustrates attempts to unify all EU member states into a single market and leaves many countries competing against one another for a better offer, for the overall number of vaccine doses distributed or for the right of first choice. Maintaining a high level of transparency is crucial to reinforce trust in the overall handling of the pandemic by the EU and by every national government, to ensure confidence in vaccines and to minimize skepticism, doubts, and suspicion. In addition, a lack of transparency may increase the risk of corruption. In this regard, António Guterres, the secretary-general of the United Nations, reported in a statement that the COVID-19 pandemic is creating new opportunities for corruption, and inadequate transparency may further increase such a risk (21). Transparency in negotiations as well as equity in global health issues should return to represent priority issues for both the EU and WHO, to avoid deplorable asymmetries in access to information, proliferation of bilateral APAs, entrenching nationalism, and directing future vaccine distribution, especially during the negotiations for the most profitable business ever: the one of COVID-19 vaccines (Figure 1). Full transparency in negotiations with the pharmaceutical companies will contribute to guarantee the success of the EU's mass COVID-19 vaccination campaign.

## Author Contributions

SS wrote the article and AB revised the text. All authors contributed to the article and approved the submitted version.

## Conflict of Interest

The authors declare that the research was conducted in the absence of any commercial or financial relationships that could be construed as a potential conflict of interest.

## Footnotes

^1^http://ec.europa.eu/health/expert_panel/index_en.htm

^2^https://agencia.fiocruz.br/sites/agencia.fiocruz.br/files/u34/contrato_etec.pdf

^3^https://epha.org/wp-content/uploads/2020/12/jointtransparency-statement-final.pdf

^4^https://www.federalregister.gov/documents/2019/11/27/2019-24931/medicare-and-medicaid-programs-cy-2020-hospital-outpatient-pps-policy-changes-and-paymentrates-and
